# Post-EV71 vaccination outcomes in meningoencephalitis: no increased clinical severity compared with other enterovirus subtypes in a single-center retrospective study

**DOI:** 10.3389/fcimb.2026.1786624

**Published:** 2026-05-12

**Authors:** Yonghan Luo, Yan Guo, Xiqi Liao, Ying Zhu, Yanchun Wang

**Affiliations:** 1 Second Department of Infectious Disease, Children’s Hospital Affiliated to Kunming Medical University (Kunming Children’s Hospital), Kunming, Yunnan, China; 2 Department of Reproductive Gynecology, NHC Key Laboratory of Healthy Birth and Birth Defect Prevention in Western China, First People’s Hospital of Yunnan Province, Kunming, Yunnan, China; 3 Department of Reproductive Gynecology, The Affiliated Hospital of Kunming University of Science and Technology, Kunming, Yunnan, China; 4 Department of Pediatrics, West China Second University Hospital, Sichuan University, Chengdu, China; 5 Key Laboratory of Birth Defects and Related Diseases of Women and Children, Sichuan University, Ministry of Education, Chengdu, China

**Keywords:** clinical outcomes, enterovirus, EV71, inflammatory markers, meningoencephalitis, vaccination

## Abstract

**Objective:**

To assess clinical differences and prognosis between pediatric meningoencephalitis caused by EV71 and other enterovirus subtypes in the context of widespread EV71 vaccination, and to evaluate changes in the clinical profile of EV71 infection in the vaccine era.

**Methods:**

We enrolled 160 children with enterovirus-associated meningoencephalitis admitted to Kunming Children’s Hospital between January 2019 and August 2024. Patients were categorized into an EV71 group and an EV-others group based on etiology. Demographic, clinical, laboratory, and outcome data were collected.

**Results:**

Of the 160 cases, 75 (46.0%) were EV-unclassified, 45 (27.6%) EV-A6, 20 (12.3%) EV71, 14 (8.6%) EV-A16, and 9 (5.5%) EV-A10. No significant between-group differences in sex, age, or weight were found. The EV71 group had a longer duration of fever (median 3.5 days vs 2 days, P = 0.032) and a higher frequency of hand–foot vesicular lesions (20/20, 100% vs 99/140, 71%, P = 0.011). Laboratory results showed higher platelet counts and lymphocyte counts in the EV71 group. In contrast, CRP, ferritin, ALT, and CD16 + 56+ levels were lower in the EV71 group. CSF analysis showed significantly higher cell counts (55.5 vs 12 ×10^6^/L, P < 0.001) in the EV71 group. There were no significant differences in ICU admission, length of hospital stays, or mortality.

**Conclusion:**

In the era of widespread EV71 vaccination, pediatric meningoencephalitis caused by EV71 no longer presents worse overall outcomes than other enterovirus subtypes. EV71 remains associated with a stronger central but weaker systemic inflammatory response, suggesting a distinct inflammatory profile. Continued surveillance and multivalent vaccine development are necessary.

## 1 Introduction

Enteroviruses belong to the family *Picornaviridae* and comprise multiple viral subtypes (Sinclair and Omar, 2025). Their impact on children is primarily reflected in the wide spectrum of infections and diseases they can cause, most notably hand, foot, and mouth disease (HFMD). Although HFMD resulting from enteroviral infection is generally a self-limiting illness, a subset of cases may progress to severe complications, particularly neurological involvement, leading to meningoencephalitis—the most serious consequence of infection (Gonzalez et al., 2019; Zhu et al., 2023).

Enteroviruses are the most common causes of viral aseptic meningitis in children, which is typically a self-limiting and relatively benign condition. However, certain enterovirus subtypes, particularly EV71, are more frequently associated with severe neurological involvement such as encephalitis and brainstem encephalitis. Meningoencephalitis represents one of the most severe complications associated with enteroviral infection. This condition can cause serious neurological damage and may lead to long-term sequelae in children, such as cognitive delay, epilepsy, hemiparesis, or even death (Tao et al., 2014; Gonzalez et al., 2019; Li et al., 2020; Ndiaye et al., 2024). Owing to its high mortality and disability rates, enterovirus-associated meningoencephalitis has emerged as a major global public health concern.

Among the various enteroviruses, EV71 is recognized as a predominant pathogen responsible for severe neurological disease in children (Solomon et al., 2010; Nayak et al., 2022). EV71 has caused outbreaks in multiple regions and has been associated with substantial pediatric morbidity and mortality (Kinobe et al., 2022). For instance, in 2008, a large-scale outbreak of HFMD occurred in Fuyang, Anhui Province, China, with a total of 6,049 reported cases. Approximately 50% of the patients required hospitalization, 5.8% developed severe illness, and the case-fatality rate reached 0.36%, posing a serious threat to the health of local children (Zhang et al., 2010).

To mitigate this threat, mainland China initiated large-scale EV71 vaccination programs beginning in 2016. Evidence from published studies demonstrates marked reductions in EV71-related morbidity and mortality following vaccine implementation (Hong et al., 2022). The systematic review showed that the EV71 inactivated vaccine is effective, with real-world effectiveness of about 67% for one dose and 84% for two doses (Liu et al., 2025). These findings underscore the critical role of EV71 vaccination in preventing severe enterovirus-associated neurological disease in children.

However, recent epidemiological data (Liu et al., 2025) indicate that although the prevalence of EV71 has declined, other enterovirus subtypes continue to cause meningoencephalitis in children. The clinical outcomes of these infections appear comparable to the sporadic EV71 cases that still occur. Nevertheless, this phenomenon has been insufficiently documented in the literature. Therefore, the present study aims to compare the clinical outcomes of meningoencephalitis caused by EV71 and other enterovirus subtypes, to examine shifts in EV71 infection patterns following vaccine introduction, and to evaluate the impact of different enteroviral subtypes on disease prognosis. The findings are intended to provide evidence to support future clinical management and inform public health strategies.

## 2 Materials and methods

### 2.1 Study population

A total of 160 cases diagnosed with enteroviral meningoencephalitis were enrolled at Kunming Children’s Hospital between January 2019 and August 2024. This study was approved by the Ethics Committee of Kunming Children’s Hospital (2025-03-316-k01).

### 2.2 Inclusion and exclusion criteria

#### 2.2.1 Inclusion criteria

1. Hospitalized patients younger than 18 years.
2. Cases clinically suspected of viral meningoencephalitis, with diagnostic criteria based on *Zhufutang Practical Pediatrics* (8th edition) (Jiang et al., 2015).

#### 2.2.2 Exclusion criteria

1. Cases confirmed to involve non-enteroviral pathogens.
2. Cases diagnosed with hand, foot, and mouth disease without definitive etiological evidence.
3. Cases in which lumbar puncture was not performed.
4. Cases in which enteroviral infection was not the primary cause of hospitalization.
5. Cases without clinical, cerebrospinal fluid, or neuroimaging findings suggestive of central nervous system infection were excluded.

Cases with etiological confirmation of EV-71 were categorized into the EV-71 group, whereas all remaining enterovirus-positive cases were classified as the EV-others group.

### 2.3 Study variables and data extraction

Data were extracted from electronic medical records and included baseline demographic information such as age, sex, and weight. Clinical symptoms collected comprised Fever, Fever duration, Disease course, Pharyngeal vesicular lesions, Hand-foot vesicular lesions, Buttocks and/or trunk vesicular lesions, Cough, Sneezing/rhinorrhea, Nasal obstruction, Headache, Vomiting, Abdominal pain, Inappetence, Diarrhea, Poor mental status, Lethargy, Irritability, Shivering, Neck rigidity, and Seizures, as well as Seizure Frequency. Laboratory tests included white blood cell count (WBC), hemoglobin (Hb), platelet count (PLT), neutrophil and lymphocyte counts, C-reactive protein (CRP), procalcitonin (PCT), interleukin-6 (IL-6), ferritin, erythrocyte sedimentation rate (ESR), immunoglobulin levels (IgA, IgG, IgM), liver function markers (ALT, AST, ALB, TBil), renal function (SCr), cardiac enzyme CKMB, coagulation parameters including APTT, PT, and fibrinogen (FG), and cerebrospinal fluid (CSF) results, including CSF total cell count, CSF glucose, CSF Chloride, and CSF protein. Immunological indices collected included CD3+, CD3+CD4+, CD3+CD8+, CD19+, CD16 + 56+, and the CD4+/CD8+ ratio. Outcome variables included Fever resolution time, Length of hospital stay, ICU admission, and Death.

In this study, the term “enterovirus-associated meningoencephalitis” was used as a broad category of enterovirus-related central nervous system infections, including meningitis, encephalitis, and meningoencephalitis. Enterovirus infection was confirmed using RT-PCR detection of enterovirus RNA in cerebrospinal fluid samples or throat swabs targeting the conserved 5′ untranslated region (5′UTR) of the enterovirus genome. Subtype identification was performed using subtype-specific PCR assays for EV71, EV-A6, EV-A10, and EV-A16. Cases that tested positive for enterovirus by the universal enterovirus PCR assay but were negative for these subtype-specific assays were classified as enterovirus-positive but subtype-unclassified infections(“EV- unclassified”).

### 2.4 Statistical analysis

Statistical analyses were performed using R software (version 4.4.1), with data visualization generated through the “ggplot” package in R. Continuous variables following a normal distribution were presented as mean ± standard deviation and compared using independent-sample t-tests. Non-normally distributed variables were reported as median (interquartile range) and evaluated with the Mann–Whitney U test. Categorical data were summarized as counts and percentages, with group differences assessed by the chi-square test. A two-sided P value < 0.05 was considered statistically significant.

## 3 Results

### 3.1 Base information

**Figure 1:**
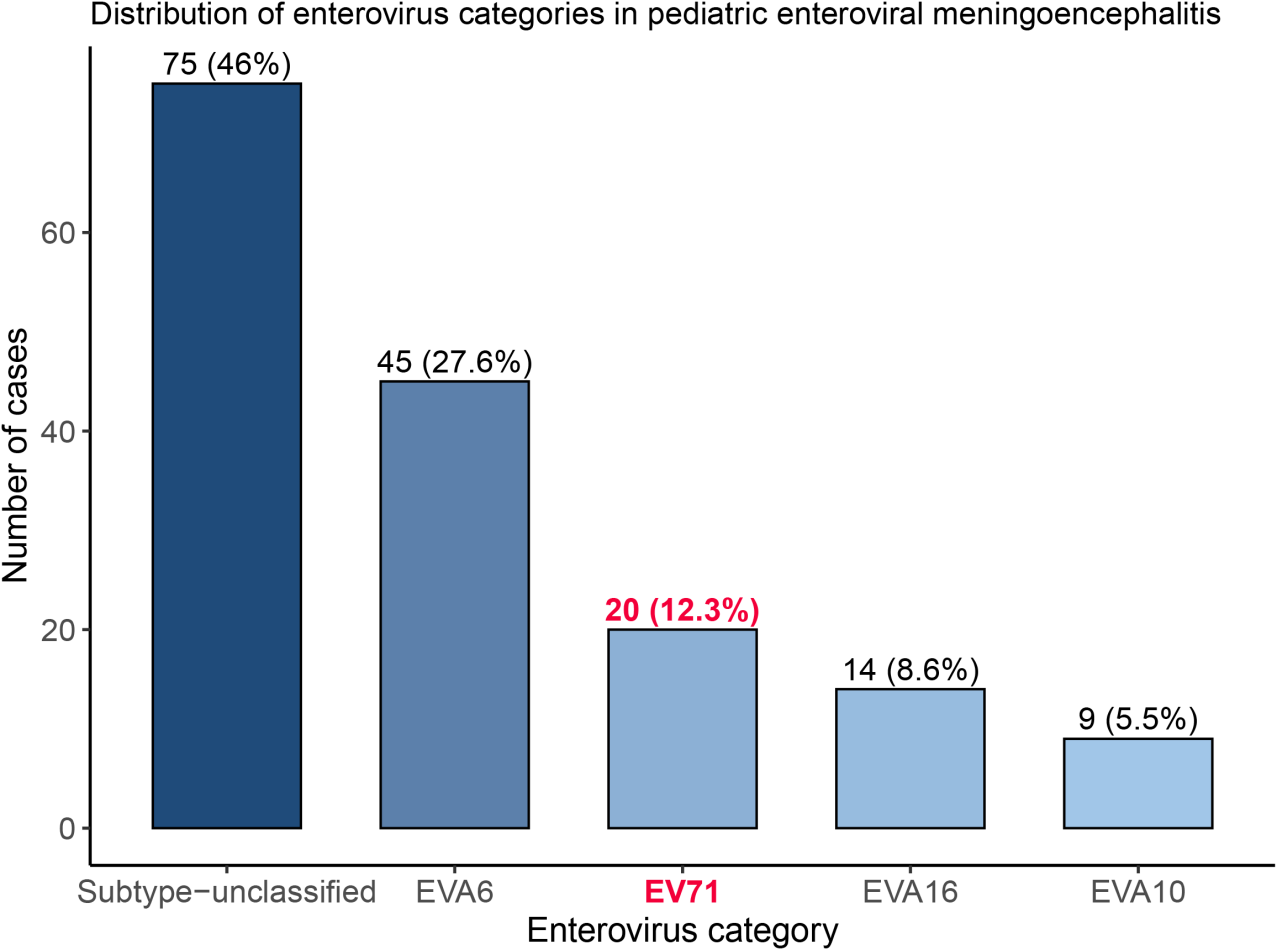
Distribution of subtypes of enterovirus meningoencephalitis.

**Figure 2:**
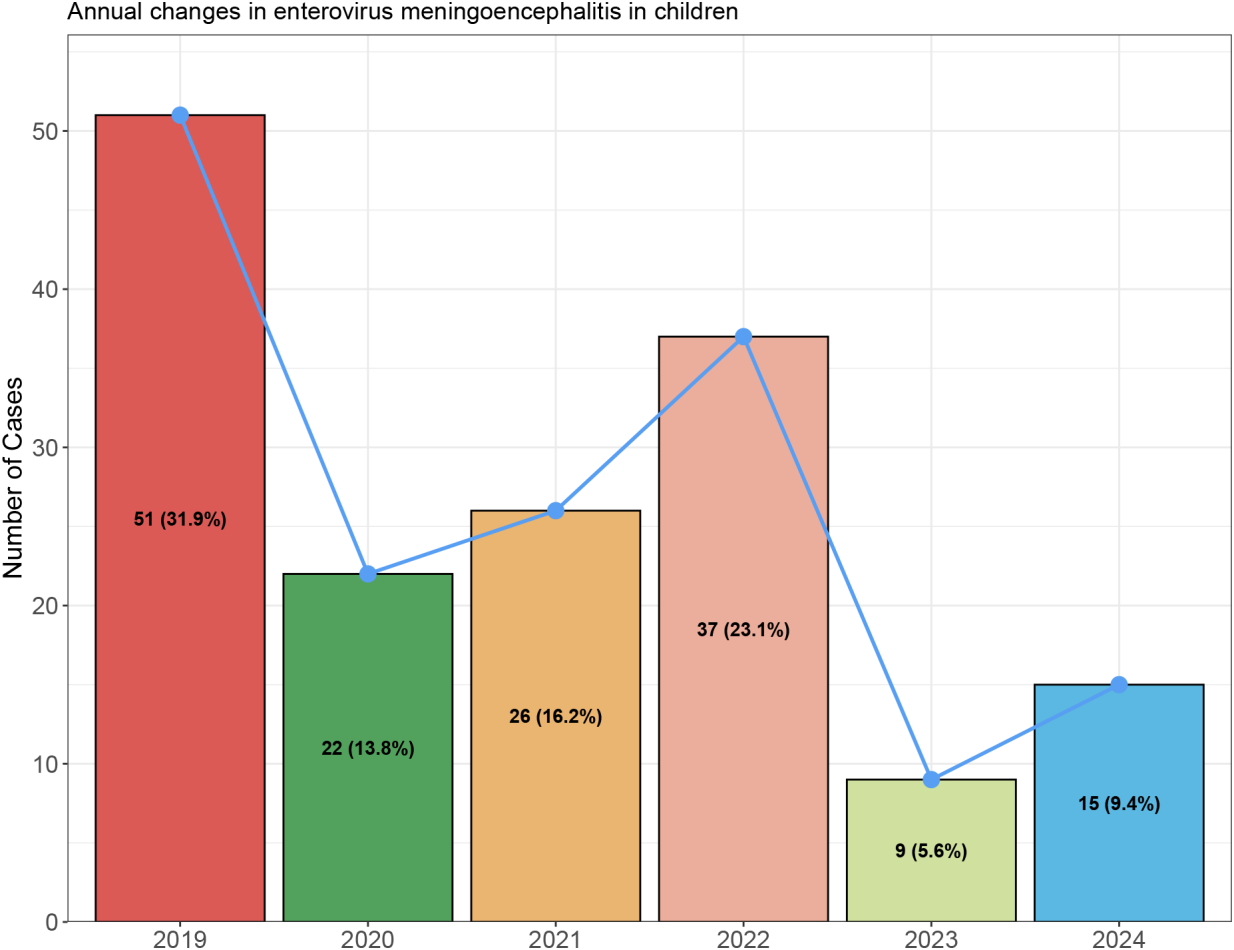
Annual changes in enterovirus meningoencephalitis.

A total of 160 cases of enterovirus meningitis were included in this study. Among these, enterovirus-unclassified cases were the most prevalent, accounting for 46.0% (75/160) of the cohort, followed by EV-A6 (27.6%, 45/160). EV71 accounted for 12.3% (20/160) of cases, while EV-A16 and EV-A10 accounted for 8.6% (14/160) and 5.5% (8/160), respectively (**Figure 1**). From 2019 to 2024, the annual number of enterovirus meningoencephalitis cases showed notable fluctuations. The highest incidence occurred in 2019 with 51 cases, followed by a marked decrease in 2020 (22 cases). Case numbers rose gradually in 2021 (26 cases) and peaked again in 2022 (37 cases). A sharp decline was observed in 2023 (9 cases), with a slight increase in 2024 (15 cases) (see **Figure 2****).**

Overall, 160 pediatric patients were enrolled, comprising 53 males (33%) and 107 females (67%). There was no significant difference in sex distribution between the two groups (P = 1.000). The median age was 2 (Q1, Q3: 1, 3) years, with no significant difference between the EV-others group and the EV-71 group (P = 0.741). The median weight was 11.95 (Q1, Q3: 10, 15) kg in the EV-others group and 11.0 (Q1, Q3: 9.73, 14.25) kg in the EV-71 group, with no statistically significant difference (P = 0.481). The baseline characteristics and symptoms of the two groups are shown in **Table 1**.

**Table 1:** Comparative baseline characteristics and symptoms of pediatric enteroviral meningoencephalitis caused by EV71 versus other enterovirus subtypes.

Characteristics	Total (n = 160)	EV-others (n = 140)	EV-71 (n = 20)	P
Baseline characteristics				
Age, Median (Q1, Q3), y	2 (1, 3)	2 (1, 3)	2 (1, 3)	0.741
Sex, n (%)				1
female	107 (67)	94 (67)	13 (65)	
male	53 (33)	46 (33)	7 (35)	
Weight, Median (Q1, Q3), kg	11.7 (10, 15)	11.95 (10, 15)	11 (9.73, 14.25)	0.481
Symptoms				
Fever, n (%)				1
No	7 (4)	6 (4)	1 (5)	
Yes	153 (96)	134 (96)	19 (95)	
Fever duration, Median (Q1, Q3), d	2 (1, 4)	2 (1, 4)	3.5 (2, 4)	0.032
Disease course, Median (Q1, Q3), d	3 (1.75, 4)	2.5 (1, 4)	4 (2, 5)	0.051
Pharyngeal vesicular lesions, n (%)				0.249
No	35 (22)	33 (24)	2 (10)	
Yes	125 (78)	107 (76)	18 (90)	
Hand-foot vesicular lesions, n (%)				0.011
No	41 (26)	41 (29)	0 (0)	
Yes	119 (74)	99 (71)	20 (100)	
Buttocks and/or trunk vesicular lesions, n (%)				0.745
No	134 (84)	118 (84)	16 (80)	
Yes	26 (16)	22 (16)	4 (20)	
Cough, n (%)				1
No	68 (42)	60 (43)	8 (40)	
Yes	92 (57)	80 (57)	12 (60)	
Sneezing/rhinorrhea, n (%)				0.719
No	140 (88)	123 (88)	17 (85)	
Yes	20 (12)	17 (12)	3 (15)	
Nasal obstruction, n (%)				1
No	148 (92)	129 (92)	19 (95)	
Yes	12 (8)	11 (8)	1 (5)	
Headache, n (%)				1
No	142 (89)	124 (89)	18 (90)	
Yes	18 (11)	16 (11)	2 (10)	
Vomiting, n (%)				0.408
No	120 (75)	103 (74)	17 (85)	
Yes	40 (25)	37 (26)	3 (15)	
Abdominal pain, n (%)				1
No	158 (99)	138 (99)	20 (100)	
Yes	2 (1)	2 (1)	0 (0)	
Inappetence, n (%)				0.22
No	145 (91)	125 (89)	20 (100)	
Yes	15 (9)	15 (11)	0 (0)	
Diarrhea, n (%)				0.557
No	154 (96)	135 (96)	19 (95)	
Yes	6 (4)	5 (4)	1 (5)	
Poor mental status, n (%)				0.688
No	146 (91)	128 (91)	18 (90)	
Yes	14 (9)	12 (9)	2 (10)	
Lethargy, n (%)				0.138
No	140 (88)	125 (89)	15 (75)	
Yes	20 (12)	15 (11)	5 (25)	
Irritability, n (%)				0.056
No	99 (62)	91 (65)	8 (40)	
Yes	61 (38)	49 (35)	12 (60)	
Shivering, n (%)				0.026
No	154 (96)	137 (98)	17 (85)	
Yes	6 (4)	3 (2)	3 (15)	
Neck rigidity, n (%)				0.204
No	132 (82)	113 (81)	19 (95)	
Yes	28 (18)	27 (19)	1 (5)	
Seizures, n (%)				0.108
0	116 (72)	98 (70)	18 (90)	
1	44 (28)	42 (30)	2 (10)	
Seizure Frequency, Median (Q1, Q3), times	2 (1, 2)	2 (1, 2)	1.5 (1.25, 1.75)	0.693

### 3.2 Comparison of symptoms and signs

**Figure 3:**
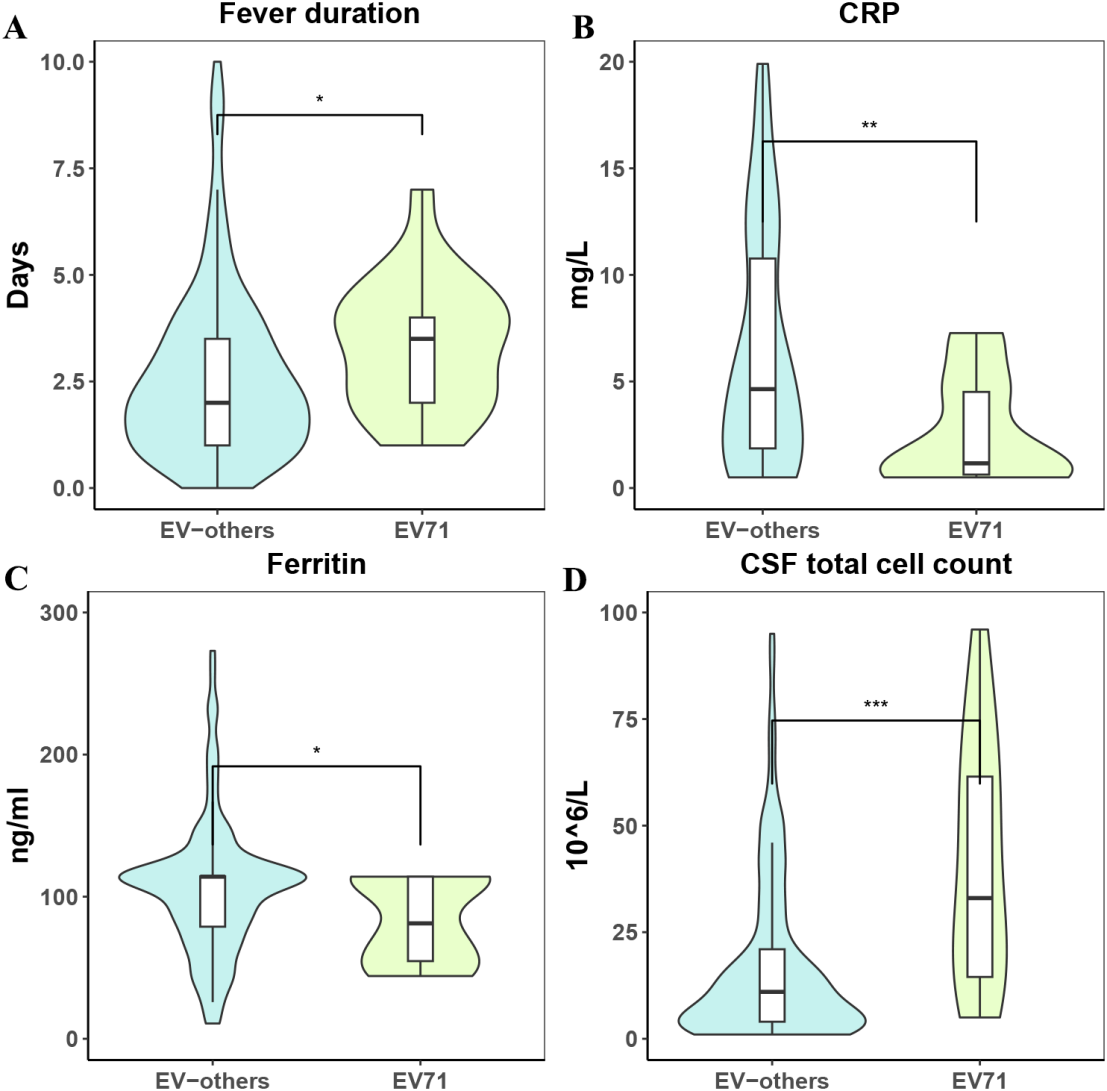
Comparison of clinical parameters between EV71 and other enteroviruses. **(A)** Fever duration: The duration of fever in the EV71 group was significantly shorter than in the EV-others group (p < 0.05). **(B)** C-reactive protein (CRP): The CRP levels in the EV71 group were significantly lower than those in the EV-others group (p < 0.01). **(C)** Ferritin: The EV71 group had lower ferritin levels compared to the EV-others group (p < 0.05). **(D)** CSF total cell count: The total cell count in the cerebrospinal fluid (CSF) was significantly higher in the EV71 group than in the EV-others group (p < 0.001).

Fever was the predominant symptom in both groups, and the presence of Fever showed no statistical difference (P = 1.000). However, Fever duration was significantly shorter in the EV-71 group〔median 3.5 days vs. 2 days, P = 0.032〕(**Figure 3A**). Disease course showed a tendency toward being longer in the EV-71 group, with a borderline significance (P = 0.051).

Regarding mucocutaneous findings, Hand-foot vesicular lesions were significantly more common in the EV-71 group (100% vs. 71%, P = 0.011). Pharyngeal vesicular lesions and Buttocks and/or trunk vesicular lesions showed no statistically significant differences between the two groups (P = 0.249 and P = 0.745, respectively).

For neurological and systemic symptoms, Shivering occurred more frequently in the EV-71 group (P = 0.026). Although not statistically significant, Irritability (P = 0.056) and Lethargy (P = 0.138) tended to be more frequent in the EV-71 group. No significant differences were observed in Headache, Vomiting, Neck rigidity, or Seizures between the two groups.

### 3.3 Laboratory tests

In hematological parameters, PLT levels were significantly higher in the EV-71 group compared with the EV-others group (367.25 vs. 314.53 ×10^9^/L, P = 0.025). Both values are within normal range. Lymphocyte count was also higher in the EV-71 group (P = 0.048).

Regarding inflammatory markers, CRP levels were significantly lower in the EV-71 group〔1.16 vs. 10.37 mg/L, P < 0.001〕(**Figure 3B**). Ferritin levels were also significantly lower in the EV-71 group (P = 0.021) (**Figure 3C****).**

With respect to liver function, ALT levels were significantly lower in the EV-71 group (11 vs. 15 U/L, P = 0.008). Both values are within normal range.

Immunological testing showed that CD16 + 56+ levels were significantly lower in the EV-71 group (P = 0.007), while CD3+, CD3+CD4+, CD3+CD8+, CD19+, and CD4+/CD8+ showed no significant differences.

In cerebrospinal fluid analysis, CSF total cell count was markedly higher in the EV-71 group〔55.5 vs. 12 ×10^6^/L, P < 0.001〕. CSF glucose, CSF Chloride, and CSF protein did not differ significantly between the two groups (**Figure 3D****).**

### 3.4 Outcomes

Fever resolution time was significantly longer in the EV-71 group〔4 vs. 3 days, P = 0.003〕. Length of hospital stay showed no significant difference between groups (P = 0.243). .

ICU admission was less frequent in the EV-71 group (5% vs. 16%), but the difference was not statistically significant (P = 0.312). Death showed no difference between groups (P = 1.000). The laboratory tests and outcomes of the two groups are shown in **Table 2**.

**Table 2:** Comparative laboratory tests and outcomes of pediatric enteroviral meningoencephalitis caused by EV71 versus other enterovirus subtypes.

Characteristics	Total (n = 160)	EV-others (n = 140)	EV-71 (n = 20)	P
Laboratory Tests				
WBC, Median (Q1, Q3), ×10^9^/L	9.91 (7.62, 12.68)	9.97 (7.9, 13.19)	9.13 (7.32, 11.57)	0.263
Hb, Median (Q1, Q3), g/L	125.47 ± 11.07	125.16 ± 11.4	127.6 ± 8.3	0.253
PLT, Median (Q1, Q3) ×10^9^/L	321.12 ± 101.95	314.53 ± 101.94	367.25 ± 91.64	0.025
Neutrophil count, Median (Q1, Q3) ×10^9^/L	5.77 (3.26, 8.87)	6.25 (3.26, 9.21)	4.64 (3.26, 6.2)	0.115
Lymphocyte count, Median (Q1, Q3) ×10^9^/L	2.79 (1.9, 3.82)	2.65 (1.79, 3.82)	3.14 (2.66, 3.96)	0.048
CRP, Median (Q1, Q3), mg/L	7.13 (2.03, 20.75)	10.37 (3.18, 28.27)	1.16 (0.63, 4.51)	< 0.001
PCT, Median (Q1, Q3), mg/L	0.25 (0.25, 0.63)	0.25 (0.25, 0.77)	0.25 (0.25, 0.26)	0.112
IL-6, Median (Q1, Q3), pg/mL	7.23 (1.5, 8.34)	7.23 (1.5, 8.34)	6.52 (2.31, 8.34)	0.983
Ferritin, Median (Q1, Q3), ng/ml	114.08 (77.25, 114.08)	114.08 (79.52, 117.5)	81.2 (54.66, 114.08)	0.021
ESR, Median (Q1, Q3), mm/h	22.56 (15, 28)	22.56 (15, 28)	19 (14.75, 23.5)	0.377
IgA, Median (Q1, Q3), g/L	0.5 (0.28, 0.77)	0.5 (0.26, 0.77)	0.52 (0.36, 0.75)	0.58
IgG, Median (Q1, Q3), g/L	7.01 (5.65, 7.99)	7.01 (5.42, 7.92)	7.01 (5.99, 8.08)	0.522
IgM, Median (Q1, Q3), g/L	1.23 (0.9, 1.57)	1.21 (0.87, 1.56)	1.38 (1.22, 1.68)	0.088
ALT, Median (Q1, Q3), U/L	15 (11, 19)	15 (11.75, 19)	11 (9, 14.28)	0.008
AST, Median (Q1, Q3), U/L	33 (28, 39.7)	33.25 (28, 39.7)	30.5 (22, 38.5)	0.159
ALB, Median (Q1, Q3), g/L	41.2 (37.65, 43.35)	41.1 (37.35, 43.35)	42.7 (39.6, 44.8)	0.151
CD3+, Median (Q1, Q3), %	51.99 (48.21, 56.15)	51.99 (48.16, 56)	52.53 (51.04, 57.84)	0.297
CD3+CD4+, Median (Q1, Q3), %	27.29 (21.87, 29.94)	27.29 (21.87, 29.13)	27.29 (23.21, 33.88)	0.372
CD3+CD8+, Median (Q1, Q3), %	19.8 (17.3, 21.09)	19.8 (17.58, 20.8)	19.8 (16.24, 24.61)	0.476
CD19+, Median (Q1, Q3), %	29.56 (26.51, 34.51)	29.56 (26.43, 33.31)	31.98 (26.96, 38.37)	0.232
CD16 + 56+, Median (Q1, Q3), %	17.39 (11.76, 18.32)	17.39 (12.53, 19.94)	11.59 (8.18, 17.39)	0.007
CD4+/CD8+, Median (Q1, Q3), %	1.55 (1.12, 1.66)	1.55 (1.12, 1.64)	1.44 (1.12, 1.78)	0.855
TBil, Median (Q1, Q3), umol/L	8.6 (6.25, 9.93)	8.55 (6.07, 9.83)	8.61 (7.35, 10.33)	0.47
SCr, Median (Q1, Q3), umol/L	21.77 (17.26, 26.22)	21.77 (18, 26.07)	20.88 (16.23, 27)	0.665
CKMB, Median (Q1, Q3), U/L	20 (17, 24)	20 (17, 24)	20 (15.75, 25.4)	0.768
APTT, Median (Q1, Q3), s	35.99 (33.55, 38.9)	35.99 (33.25, 38.68)	35.99 (35.4, 39.6)	0.998
PT, Median (Q1, Q3), s	13.4 (12.5, 14.7)	13.4 (12.47, 14.7)	14.05 (12.65, 14.7)	0.466
FG, Median (Q1, Q3), g/L	3.79 (3.2, 3.94)	3.79 (3.2, 4.06)	3.7 (3.27, 3.79)	0.083
CSF total cell count, Median (Q1, Q3),10^6^/L	14 (5, 42)	12 (4, 31.75)	55.5 (24.75, 97.5)	< 0.001
CSF glucose, Median (Q1, Q3), mmol/L	3.7 (3.37, 4.21)	3.74 (3.38, 4.24)	3.6 (3.36, 3.8)	0.184
CSF Chloride, Median (Q1, Q3), mmol/L	125 (123.47, 127.2)	125 (123.6, 127.25)	125 (122.85, 126.25)	0.501
CSF protein, Median (Q1, Q3), g/L	0.1 (0.05, 0.19)	0.1 (0.05, 0.17)	0.16 (0.1, 0.21)	0.062
Outcomes				
Fever resolution time, Median (Q1,Q3), d	3 (2, 4)	3 (2, 4)	4 (3.75, 4.25)	0.003
Length of hospital stay, Median (Q1,Q3), d	7 (6, 8)	7 (6, 7)	7 (6, 8)	0.243
ICU admission, n (%)				0.312
No	137 (86)	118 (84)	19 (95)	
Yes	23 (14)	22 (16)	1 (5)	
Death, n (%)				1
No	158 (99)	138 (99)	20 (100)	
Yes	2 (1)	2 (1)	0 (0)	

WBC, white blood cell count; Hb, hemoglobin; PLT, platelet count; CRP, C-reactive protein; PCT, procalcitonin; IL-6, interleukin-6; ESR, erythrocyte sedimentation rate; IgA, immunoglobulin A; IgG, immunoglobulin G; IgM, immunoglobulin M; ALT, alanine aminotransferase; AST, aspartate aminotransferase; ALB, albumin; CD3+, cluster of differentiation 3 positive; CD3+CD4+, cluster of differentiation 3 positive, cluster of differentiation 4 positive; CD3+CD8+, cluster of differentiation 3 positive, cluster of differentiation 8 positive; CD19+, cluster of differentiation 19 positive; CD16 + 56+, cluster of differentiation 16 positive, cluster of differentiation 56 positive; CD4+/CD8+, ratio of cluster of differentiation 4 positive to cluster of differentiation 8 positive; TBil, total bilirubin; SCr, serum creatinine; CKMB, creatine kinase-MB; APTT, activated partial thromboplastin time; PT, prothrombin time; FG, fibrinogen; ICU, Intensive Care Unit; CSF, Cerebrospinal Fluid.

## 4 Discussion

This retrospective study of 160 pediatric enterovirus meningoencephalitis cases from 2019–2024 yielded three main findings. First, EV71-associated meningoencephalitis no longer resulted in poorer outcomes than other subtypes, with comparable hospital stay, ICU admission, and mortality. Second, EV71 accounted for only 12.3% of cases, confirming its epidemiological decline following vaccination. Third, EV71 exhibited a distinct inflammatory profile: stronger central nervous system inflammation (higher CSF cell counts) but milder systemic responses (lower CRP and ferritin). These findings suggest that, in the post-vaccination era, EV71-associated meningoencephalitis does not lead to worse clinical outcomes while retaining a distinct inflammatory signature.

Comparison with pre-vaccination era studies further contextualizes these findings. Prior to widespread EV71 vaccination in China, EV71 was the predominant cause of severe neurological disease and was associated with substantially worse outcomes. During the 2008 Fuyang outbreak, EV71 was identified as the primary causative agent: among 6,049 cases, 3,023 (50%) required hospitalization, 353 (5.8%) were classified as severe, and 22 (0.36%) were fatal (Zhang et al., 2010), and studies from the pre-vaccine era consistently reported higher rates of brainstem encephalitis, neurogenic pulmonary edema, and mortality in EV71-infected children (Solomon et al., 2010; Majer et al., 2020). In stark contrast, our post-vaccination cohort demonstrates not only a reduced proportion of EV71 cases (12.3%) but also the absence of EV71-attributable mortality and severe outcomes comparable to other enteroviruses. These comparisons strongly suggest that widespread EV71 vaccination has fundamentally altered the clinical landscape of enterovirus meningoencephalitis, transforming EV71 from a highly neurovirulent pathogen into one with clinical severity similar to other circulating enterovirus subtypes.

The genus Enterovirus comprises multiple species (Enterovirus A-D), which encompass a wide array of serotypes with varying capacities to invade the central nervous system (CNS) and elicit distinct clinical syndromes (Majer et al., 2020). For instance, while many enteroviruses can cause aseptic meningitis—the most common CNS manifestation—certain serotypes exhibit stronger neurotropism. Polioviruses (Enterovirus C species) are classically associated with acute flaccid paralysis due to their destruction of anterior horn cells. Among the non-polio enteroviruses, EV71 (Enterovirus A species) is well-documented for its propensity to cause severe brainstem encephalitis and neurogenic pulmonary edema. Other serotypes, such as Echoviruses and certain coxsackieviruses (e.g., CV-B5), are frequently implicated in meningitis but less commonly in severe encephalitis (Ong and Wong, 2015). This study focuses on meningoencephalitis, and within this context, we specifically examined the evolving role of EV71 in the post-vaccination era.

Within the diverse enterovirus genus, the pathogenic mechanisms leading to CNS disease can vary. Upon entering the host via the respiratory or gastrointestinal tract, enteroviruses typically replicate in lymphatic tissues, leading to viremia and potential CNS invasion (Jiang et al., 2015). The resultant meningoencephalitis involves direct viral neuronal injury, immune-mediated inflammatory damage, and cytokine-storm–associated cerebral edema. The clinical syndrome that emerges depends on both the viral serotype and the affected CNS regions. As noted above, EV71 exhibits pronounced neurotropism for the brainstem. It traverses the blood–brain barrier and preferentially involves the medulla oblongata and pons, leading to “brainstem encephalitis.” This specific localization is closely associated with severe autonomic dysregulation, sympathetic storm, and the life-threatening complication of neurogenic pulmonary edema (Ong and Wong, 2015; Majer et al., 2020). In contrast, other enteroviruses (e.g., Echoviruses, CV-A16) more frequently cause diffuse meningitis or mild encephalitis without such focal brainstem involvement. These distinct pathogenic mechanisms underpin the historically high fatality rates associated with EV71 infection compared to other enterovirus serotypes.

Enteroviruses typically invade the host via the respiratory or gastrointestinal tract, replicate within the lymphatic system, and induce viremia, subsequently breaching the central nervous system to cause meningoencephalitis (Chen et al., 2020). Pathogenic mechanisms of enterovirus meningoencephalitis include direct viral injury to neurons, immune-mediated inflammatory damage, and cytokine-storm–associated cerebral edema. Compared with other subtypes, EV71 exhibits pronounced neurotropism; it traverses the blood–brain barrier and preferentially involves the brainstem, leading to “brainstem encephalitis,” which is closely associated with severe autonomic dysregulation, sympathetic storm, and neurogenic pulmonary edema (Wang et al., 2012; Teoh et al., 2016). These mechanisms underpinned the historically high fatality rates associated with EV71 infection.

Since the nationwide introduction of the inactivated EV71 vaccine in 2016, vaccine-induced high-titer neutralizing antibodies have effectively suppressed viral replication and curtailed its neuroinvasive potential (Li et al., 2021). Based on nationwide HFMD surveillance data from China during 2013–2019, after the monovalent EV71 vaccine was launched in 2016, HFMD mortality decreased by 83.78% and the proportion of severe cases declined by 60.7%; moreover, the risk of progression to severe disease among EV71 infections was reduced, suggesting that vaccination substantially lowered the burden of EV71–associated severe disease and death (Hong et al., 2022). Consistent with these epidemiological observations, the clinical outcomes observed in our cohort suggest that EV71 infection in children with meningoencephalitis was not associated with worse prognosis compared with other enterovirus subtypes.

Although vaccination markedly reduces the risk of severe EV71 disease, sporadic cases of EV71-positive meningoencephalitis persist, indicating low-level community circulation (Mao et al., 2016, Hu et al., 2024). While the vaccine does not fully prevent infection, it significantly mitigates disease severity. In this cohort, EV71-associated meningitis did not result in longer hospitalization, higher ICU admission, or greater mortality than infections caused by other enterovirus subtypes, and clinical trajectories remained generally stable. This may reflect rapid viral clearance and restricted central nervous system dissemination enabled by vaccine-induced immunological memory, thereby reducing the likelihood of brainstem involvement and other critical complications (Andronik et al., 2025). These findings underscore that among vaccinated children, EV71 infection typically manifests with mild presentations, alleviating unnecessary concern for both clinicians and caregivers.

This study identified distinct laboratory features within the EV71 group. Notably, EV71-infected children exhibited prolonged fever and elevated cerebrospinal fluid cell counts, which may reflect a relatively stronger central nervous system inflammatory response, consistent with the virus’s known neurotropic properties. However, given the observational design of this study, these findings should be interpreted as descriptive laboratory patterns rather than direct evidence of underlying mechanisms. Conversely, peripheral inflammatory markers such as CRP and ferritin were lower in the EV71 group than in other subtypes. This observation may indicate relatively milder systemic inflammatory activation in EV71-associated cases within this cohort. Taken together, these findings suggest a pattern of relatively stronger central but weaker systemic inflammatory responses in EV71-associated meningoencephalitis, based on the laboratory markers available in this study. This pattern should be interpreted as an observational inflammatory profile rather than a defined mechanistic phenotype. In combination with the comparable clinical outcomes observed between groups, these findings provide further descriptive evidence regarding the clinical characteristics of EV71-associated meningoencephalitis in the post-vaccination era.

With increasing EV71 vaccine coverage, both domestic and international surveillance data indicate that EV71 has progressively receded from dominance among circulating strains, while EV-A6, EV-A10, EV-A16, and others have emerged as the principal etiologies of recent meningitis and HFMD outbreaks (Liu et al., 2025). The viral spectrum observed in this study concurs with this epidemiological shift.

This study has several limitations. First, as a single-center retrospective analysis, it is subject to selection bias and may not fully represent broader population characteristics. Second, individualized vaccination histories and antibody titers were unavailable, precluding deeper exploration of the relationship between immune status and disease severity. Finally, viral load quantification was not performed, limiting assessment of associations between viral replication kinetics across subtypes and their corresponding clinical manifestations.

## Data Availability

The raw data supporting the conclusions of this article will be made available by the authors, without undue reservation.
